# Weight-adjusted lean body mass and calf circumference are protective against obesity-associated insulin resistance and metabolic abnormalities

**DOI:** 10.1016/j.heliyon.2017.e00347

**Published:** 2017-07-04

**Authors:** Toshinari Takamura, Yuki Kita, Masatoshi Nakagen, Masaru Sakurai, Yuki Isobe, Yumie Takeshita, Kohzo Kawai, Takeshi Urabe, Shuichi Kaneko

**Affiliations:** a Department of Endocrinology and Metabolism, Kanazawa University Graduate School of Medical Sciences, Ishikawa, Japan; b Department of System Biology, Kanazawa University Graduate School of Medical Sciences, Ishikawa, Japan; c Social and Environmental Medicine, Kanazawa Medical University, Ishikawa, Japan; d Department of Internal Medicine, Public Central Hospital of Matto Ishikawa, Hakusan, Ishikawa, Japan

**Keywords:** Health sciences, Biological sciences, Medicine, Metabolism, Endocrinology, Anatomy

## Abstract

**Background:**

To test the hypothesis that preserved muscle mass is protective against obesity-associated insulin resistance and metabolic abnormalities, we analyzed the relationship of lean body mass and computed tomography-assessed sectional areas of specific skeletal muscles with insulin resistance and metabolic abnormalities in a healthy cohort.

**Methods:**

A total of 195 subjects without diabetes who had completed a medical examination were included in this study. Various anthropometric indices such as circumferences of the arm, waist, hip, thigh, and calf were measured. Body composition (fat and lean body mass) was determined by bioelectrical impedance analysis. Sectional areas of specific skeletal muscles (iliopsoas, erector spinae, gluteus, femoris, and rectus abdominis muscles) were measured using computed tomography.

**Findings:**

Fat and lean body mass were significantly correlated with metabolic abnormalities and insulin resistance indices. When adjusted by weight, relationships of fat and lean body mass with metabolic parameters were mirror images of each other. The weight-adjusted lean body mass negatively correlated with systolic and diastolic blood pressures; fasting plasma glucose, HbA1c, alanine aminotransferase, and triglyceride, and insulin levels; and hepatic insulin resistance indices, and positively correlated with HDL-cholesterol levels and muscle insulin sensitivity indices. Compared with weight-adjusted lean body mass, weight-adjusted sectional areas of specific skeletal muscles showed similar, but not as strong, correlations with metabolic parameters. Among anthropometric measures, the calf circumference best reflected lean body mass, and weight-adjusted calf circumference negatively correlated with metabolic abnormalities and insulin resistance indices.

**Interpretation:**

Weight-adjusted lean body mass and skeletal muscle area are protective against weight-associated insulin resistance and metabolic abnormalities. The calf circumference reflects lean body mass and may be useful as a protective marker against obesity-associated metabolic abnormalities.

## 1 Introduction

Insulin resistance and its related metabolic abnormalities, such as type 2 diabetes, hypertension, and dyslipidemia, increase risks of cardiovascular diseases and cancers. Although obesity may play a major role in the development of insulin resistance, recent studies suggest that ectopic lipid accumulation in insulin-targeting organs, such as the liver [1] and skeletal muscles [2], in an overnutrition state may also contribute to the pathology of insulin resistance via perturbation of inter-organ networks mediated by nutrients, hepatokines/myokines, and neuronal pathways [3]. The skeletal muscle plays an important role in glucose uptake and energy expenditure. In the cross-sectional observations, lower relative muscle mass, that is estimated from the ratio of lean body mass to total body weight, is associated with insulin resistance [4, 5]. We recently found that lower absolute lean body is associated with basal energy expenditure and diet-induced thermogenesis [6]. In a longitudinal cohort study, greater lean body mass loss occurred in insulin-resistant men [7]. These findings suggest that loss of lean body mass may be both cause and consequence of insulin resistance. However, no study has compared lean-body mass and imaging-assessed various skeletal muscle mass comprehensively in relation to insulin resistance and metabolic abnormalities. In addition, conventional anthropometric index reflecting lean body mass is not established to date. It was reported that risks of myocardial infarction are positively correlated with the waist-to-hip ratio and waist circumference (WC) and negatively correlated with the hip circumference (HC) [8]. Because HC may be reflective of muscle mass and peripheral subcutaneous fat [6], we hypothesized that preserved muscle mass is protective against obesity-associated insulin resistance and metabolic abnormalities. In the present study, we tested this hypothesis by analyzing the relationship of lean body mass and computed tomography (CT)-assessed sectional areas of specific skeletal muscles with insulin resistance/metabolic abnormalities in a healthy cohort. In addition, we determined the anthropometric measure best reflecting lean body mass.

## 2 Materials and methods

### 2.1 Study population

**Fig. 1:**
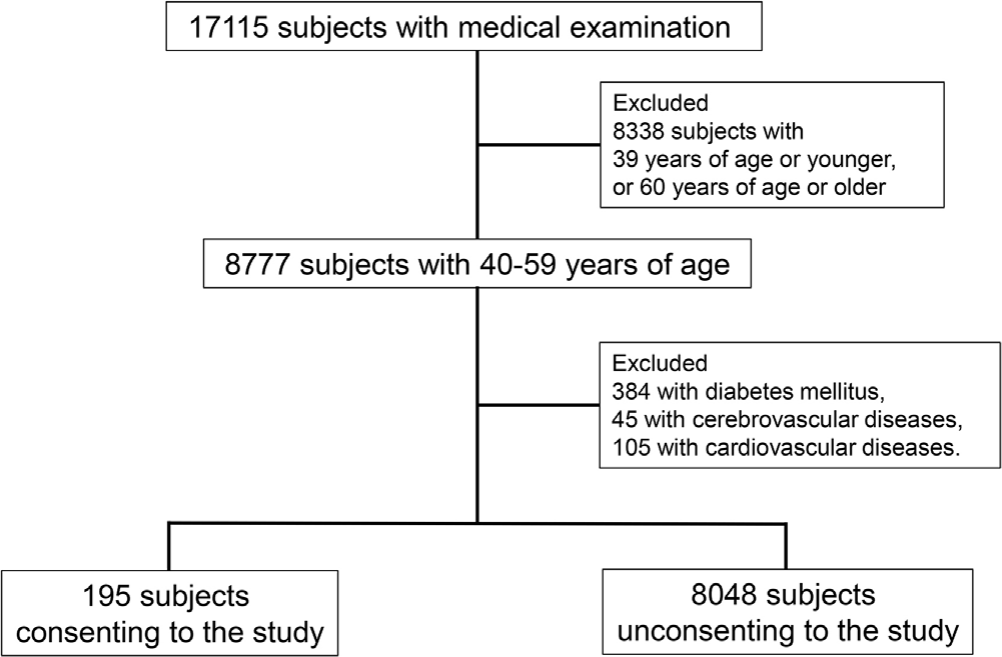
Flow diagram for subject population.

A total of 17115 subjects underwent a medical examination at the Public Central Hospital of Matto Ishikawa from 2010 to 2012 (Fig. 1). Of these, we included 8777 subjects (40–59). We excluded 384 subjects with diabetes mellitus, 45 subjects with cerebrovascular diseases, and 105 subjects with cardiovascular diseases. Of the remaining 8243 subjects, we analyzed 195 subjects who consented to participate in the present study (Fig. 1).

Clinical characteristics of the study subjects are shown in Table 1. The subjects were not taking antidiabetic agents or medications that are known to influence the primary outcome measures of this study; 17, 8, and 9 subjects were taking antihypertensive, lipid-lowering, and both antihypertensive and lipid-lowering agents, respectively.

**Table 1:** Clinical characteristics of the study subjects.

	All (n = 195)	Male (n = 88)	Female (n = 107)
Male: Female	88:107		
Age (years)	50.1 ± 5.7	50.8 ± 5.8	49.3 ± 5.6
Body weight (kg)	64.5 ± 13.2	73.3 ± 10.5	54.9 ± 8.3
Body mass index (kg/m^2^)	23.4 ± 4.0	25.0 ± 3.5	21.6 ± 3.7
Waist circumference (cm)	82.3 ±3.6	87.3 ± 9.2	76.1 ± 10.6
Systolic blood pressure (mmHg)	119.8 ± 13.7	128.0 ± 12.7	110.6 ± 17.8
Diastolic blood pressure (mmHg)	76.5 ± 17.5	82.8 ± 9.4	69.4 ± 11.4
Fasting plasma glucose (mg/dL)	96.3 ± 11.2	101.0 ± 11.7	91.4 ± 8.2
HbA1c (%)	5.6 ± 0.4	5.6 ± 0.4	5.6 ± 0.3
Creatinine (mg/dL)	0.75 ± 0.16	0.86 ± 0.12	0.63 ± 0.09
Alanine aminotransferase (IU/L)	24.5 ± 17.2	30.2 ± 20.2	18.3 ± 10.3
Total cholesterol (mg/dL)	206.4 ± 37.3	198.3 ± 26.7	215.4 ± 44.9
Triglyceride (mg/dL)	126.2 ± 106.6	156.4 ± 130.0	92.8 ± 57.7
High-density lipoprotein-cholesterol (mg/dL)	61.0 ± 16.6	54.9 ± 14.1	67.8 ± 16.7
Low-density lipoprotein-cholesterol (mg/dL)	119.2 ± 27.4	115.5 ± 25.5	123.2 ± 29.2
Serum insulin (IU/L)	5.7 ± 3.6	6.3 ± 4.2	5.0 ± 2.7

The study was conducted with the approval of the ethics committee of Kanazawa University Hospital, Ishikawa, Japan, in accordance with the Declaration of Helsinki. Written informed consent was obtained from all subjects before enrollment. This trial is registered with the University Hospital Medical Information Network (UMIN) Clinical Trials Registry, number UMIN000012630.

### 2.2 Anthropometric measures

A total of 6 professional nurses subjecting to the division of medical examination at the Public Central Hospital of Matto Ishikawa performed anthropometric measurement in the present study. BMI was calculated as weight (kg) divided by height (m) squared. We used the standardized anthropometric measurement methods for Specific Health Checkups defined by the Ministry of Health, Labor, and Welfare in Japan (The National Health and Nutrition Survey in Japan, 2004; http://www.nibiohn.go.jp/eiken/english/research/pdf/nhns2004.pdf). WC was measured at the umbilical level during quiet breathing. HC was measured at the level of the anterior superior iliac spine with the subject wearing minimal clothing and standing with his/her feet together. The arm circumference (AC), thigh circumference (TC), and calf circumference (CC) were measured on both right and left sides at mid-upper arm, at 15 cm above the upper edge of the patella, and at the greatest dimension of the calf, respectively, and the average value of both sides was calculated.

Blood pressure was measured two times consecutively using a mercury sphygmomanometer, and the lower value was used for analyses.

Body composition, including fat and lean body mass, was determined by dual-frequency bioelectrical impedance analysis (Tanita DC-320™, Tanita, Tokyo, Japan).

The sectional areas of specific skeletal muscles were measured by CT images using an image analyzing software (fatPointer™ version 2, Hitachi Medical Corporation, Tokyo, Japan); the iliopsoas, erector spinae, and rectus abdominis muscles were measured at the umbilical level; the gluteal muscle was measured at the maximal HC level; and the femoris muscle was measured 10 cm above the upper margin of the patella.

### 2.3 Biochemical parameters

Blood samples were obtained from all subjects after 8 h of fasting. Samples were immediately centrifuged, and plasma and serum samples were stored at −20 °C until analysis. Glucose levels were measured using a standard glucose oxidase method (747 Automatic Analyzer, Hitachi, Tokyo, Japan). Creatinine, alanine aminotransferase, aspartate aminotransferase, total cholesterol, high-density lipoprotein cholesterol (HDL-C), and triglyceride (TG) levels were enzymatically measured using a chemical analyzer (Daiichi, Hitachi 747, Japan). Subjects with TG levels of more than 400 mg/dL were not included in the study. Low-density lipoprotein cholesterol (LDL-C) levels were calculated according to the Friedewald formula. Fasting serum insulin levels were determined by chemiluminescence (RIA Kit, Daiichi, Japan), and glycosylated hemoglobin levels (NGSP values) were measured by immunoturbidimetry (Cobas Integra 800™, Roche, Mannheim, Germany).

### 2.4 Assessment of insulin sensitivity

Insulin resistance was estimated using the homeostasis model assessment of insulin resistance (HOMA-IR) index, calculated using the following formula: HOMA-IR = fasting glucose (mg/dL) × fasting insulin (μU/mL)/405 [9]. The quantitative insulin sensitivity check index (QUICKI), a parameter of insulin sensitivity, was calculated using the logarithmic transformation: 1/[log fasting insulin (μU/mL) + log fasting glucose (mg/dL)] [10].

After an overnight fast (10–12 h), a 75-g OGTT was performed. Blood samples were obtained at 0, 30, 60, and 120 min after the glucose load to measure plasma glucose and serum insulin levels. Insulin resistance indices were calculated using OGTT data as proposed by Matsuda and DeFronzo [11, 12]. The Matsuda index [11], an index that was shown to be strongly correlated with the rate of whole-body (mainly skeletal muscle) glucose disposal in euglycemic insulin clamp studies, was calculated using the following formula: Matsuda index = 10,000/√ (fasting glucose × fasting insulin) × (mean glucose × mean insulin during OGTT). The hepatic insulin resistance (H-IR) index [12], which is strongly correlated with H-IR in euglycemic insulin clamp studies (fasting serum insulin × basal endogenous glucose production), was defined as the product of the total areas under the curve (AUC) for glucose and insulin during the first 30 min of OGTT and was calculated using the following formula: H-IR = [AUC(glucose)0–30] × [AUC(insulin)0–30].

In any given individual, HOMA-IR and H-IR (primarily liver) indices, QUICKI, and Matsuda index (muscle plus liver) provide different information [12].

### 2.5 Statistical analyses

All results are expressed as the mean ± standard deviation. The values were converted to log values if they were not normally distributed. Intergroup comparisons were performed using ANOVA. A Pearson’s correlation analysis was also performed to evaluate the relationship of insulin resistance with metabolic parameters, including CC. Multiple linear regression analysis was performed to analyze independent associations of body composition/muscle mass with the insulin sensitivity index, or those of CC and lean body mass with the insulin sensitivity index, after adjusting for confounding factors. All calculations in the statistical analysis were performed using SPSS™ 15.0 (SPSS Inc., Chicago, IL, USA).

## 3 Results

### 3.1 Association of fat/lean body mass or computed tomography-assessed maximal sectional area of specific skeletal muscles with metabolic parameters

Univariate partial correlations of fat mass, lean body mass, or sectional area of specific skeletal muscles with metabolic parameters, adjusted by age and sex, are shown in Table 2. Fat and lean body mass were positively correlated with systolic and diastolic blood pressure and fasting plasma glucose, HbA1c, creatinine, alanine aminotransferase, TG, and insulin levels and negatively correlated with HDL-C levels. Also, fat and lean body mass were negatively correlated with muscle insulin sensitivity indices, such as the Matsuda index and QUICKI, and positively correlated with hepatic insulin resistance indices, such as HOMA-IR and H-IR.

**Table 2:** Partial correlation of fat mass, lean body mass and area of muscles with metabolic parameters.

			Fat mass		Lean body mass		Iliopsoas muscle		Erector spinae muscle		Gluteal muscle		Femoris muscle		Rectus abdominis muscle	
			r	p	r	p	r	p	r	p	r	p	r	p	r	p
Body weight (kg)			0.941	0.000	0.913	0.000	0.493	0.000	0.281	0.000	0.295	0.000	0.763	0.000	0.376	0.000
Waist circumference (cm)			0.905	0.000	0.765	0.000	0.398	0.000	0.167	0.025	0.194	0.009	0.674	0.000	0.338	0.000
Systolic blood pressure (mmHg)			0.430	0.000	0.293	0.000	0.172	0.021	0.094	0.209	0.083	0.268	0.353	0.000	0.105	0.162
Diastolic blood pressure (mmHg)			0.498	0.000	0.375	0.000	0.259	0.000	0.198	0.008	0.135	0.072	0.412	0.000	0.136	0.069
Fasting plasma glucose (mg/dL)			0.327	0.000	0.237	0.001	0.062	0.408	0.139	0.063	0.078	0.298	0.268	0.000	0.152	0.041
HbA1c (%)			0.410	0.000	0.312	0.000	0.124	0.098	0.115	0.125	0.153	0.040	0.296	0.000	0.106	0.158
Creatinine (mg/dL)			-0.161	0.031	-0.060	0.426	0.176	0.018	0.006	0.936	0.260	0.001	-0.002	0.980	0.157	0.035
Alanine aminotransferase (IU/L)			0.390	0.000	0.341	0.000	0.218	0.003	0.079	0.295	0.183	0.014	0.277	0.000	-0.008	0.920
Total cholesterol (mg/dL)			0.085	0.255	0.097	0.196	0.084	0.260	0.079	0.291	0.100	0.182	0.150	0.045	-0.002	0.978
Triglyceride (mg/dL)			0.317	0.000	0.237	0.001	0.129	0.085	0.090	0.232	0.063	0.403	0.218	0.003	0.061	0.419
High-density lipoprotein-cholesterol (mg/dL)			-0.472	0.000	0.018	0.808	-0.209	0.005	-0.102	0.175	-0.094	0.208	-0.318	0.000	-0.138	0.064
Glucose tolerance test																
	Plasma glucose (mg/dL)	0 min	0.327	0.000	0.237	0.001	0.062	0.408	0.139	0.063	0.078	0.298	0.268	0.000	0.152	0.041
		30 min	0.122	0.104	0.018	0.808	0.025	0.742	0.017	0.819	0.018	0.811	0.079	0.295	0.012	0.869
		60 min	0.316	0.000	0.165	0.027	0.058	0.442	-0.014	0.851	0.026	0.726	0.168	0.024	0.098	0.193
		120 min	0.314	0.000	0.205	0.006	0.083	0.270	0.096	0.201	0.017	0.824	0.208	0.005	0.031	0.682
	Serum insulin (IU/L)	0 min	0.572	0.000	0.336	0.000	0.195	0.009	0.085	0.255	0.033	0.661	0.308	0.000	0.015	0.845
		30 min	0.276	0.000	0.165	0.027	0.107	0.154	0.000	0.996	-0.056	0.456	0.147	0.049	-0.073	0.333
		60 min	0.387	0.000	0.178	0.017	0.061	0.415	-0.042	0.573	-0.052	0.488	0.146	0.050	-0.067	0.373
		120 min	0.431	0.000	0.175	0.019	0.106	0.155	-0.023	0.757	-0.060	0.424	0.202	0.007	-0.074	0.322
Insulinogenic index			0.150	0.044	0.149	0.046	0.041	0.588	-0.004	0.955	-0.041	0.589	0.091	0.223	-0.049	0.511
Hepatic insulin resistance index			0.362	0.000	0.209	0.005	0.125	0.095	0.035	0.645	-0.038	0.614	0.205	0.006	-0.048	0.521
Matsuda index			-0.586	0.000	-0.324	0.000	-0.165	0.026	-0.054	0.468	0.002	0.982	-0.308	0.000	0.010	0.891
HOMA-IR			0.587	0.000	0.352	0.000	0.193	0.010	0.101	0.176	0.043	0.568	0.330	0.000	0.037	0.622
QUICKI			-0.580	0.000	-0.336	0.000	-0.188	0.011	-0.090	0.231	-0.036	0.632	-0.325	0.000	-0.035	0.639
Partial correlation was analysed, adjusted with age and sex.																

The CT-assessed sectional area of the specific skeletal muscles, particularly the femoris muscle, also correlated with metabolic parameters to some extent (Table 2). However, the correlations were not as strong as those of fat/lean body mass with metabolic parameters.

### 3.2 Association of weight-adjusted fat/lean body mass or weight-adjusted computed tomography-assessed maximal sectional area of specific skeletal muscles with metabolic parameters

**Fig. 2:**
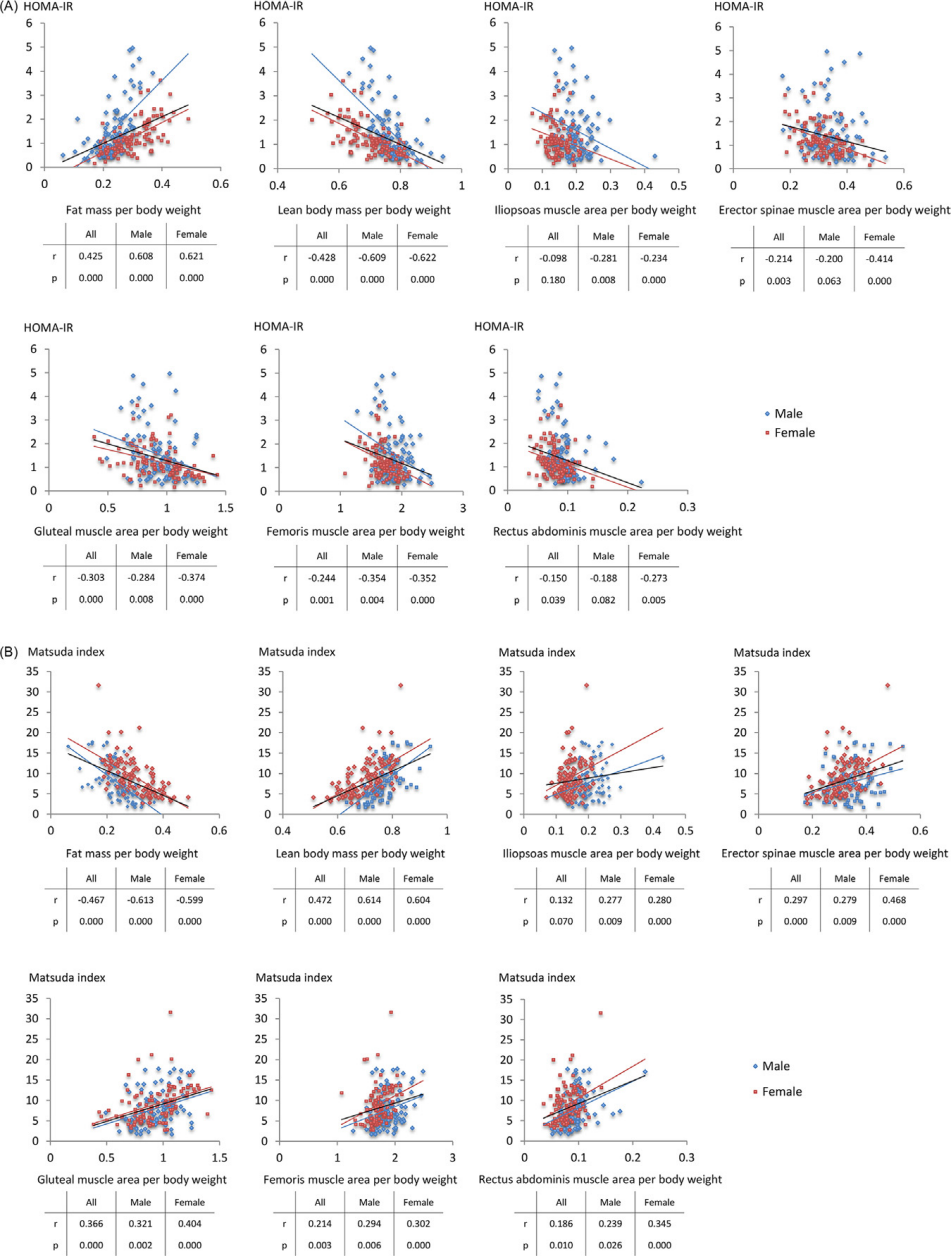
A. Scatter diagrams between insulin resistance HOMA-IR index, and lean body mass per body weight, fat mass per body weight, or specific muscle areas per body weight. B. Scatter diagrams between insulin sensitivity Matsuda index, and lean body mass per body weight, fat mass per body weight, or specific muscle areas per body weight.

Because obese people have a high fat and lean mass, we hypothesized that weight-adjusted lean body mass more accurately reflects the benefits of skeletal muscle mass against obesity-associated metabolic abnormalities. As expected, when each parameter was adjusted by weight, the relationship of fat and lean body mass with metabolic parameters became mirror images of each other (Table 3A, Fig. 2A, Fig. 2B). Weight-adjusted lean body mass was protective against obesity-associated metabolic abnormalities; it negatively correlated with systolic and diastolic blood pressures and fasting plasma glucose, HbA1c, alanine aminotransferase, TG and insulin levels and positively correlated with HDL-C levels. In addition, the weight-adjusted lean body mass was positively correlated with muscle insulin sensitivity indices, such as Matsuda index (Fig. 2B) and QUICKI, and negatively correlated with hepatic insulin resistance indices, such as HOMA-IR (Fig. 2A) and H-IR, suggesting that skeletal muscle mass to body weight ratio reflects systemic insulin sensitivity.

**Table 3A:** Partial correlation of weight-adjusted fat mass, lean body mass and sectional area of specific skeletal muscles with metabolic parameters.

		Weight-adjusted fat mass		Weight-adjusted lean body mass		Weight-adjusted iliopsoas muscle		Weight-adjusted erector spinae muscle		Weight-adjusted gluteal muscle		Weight-adjusted femoris muscle		Weight-adjusted rectus abdominis muscle	
	
		r	p	r	p	r	p	r	p	r	p	r	p	r	p
Waist circumference (cm)		0.841	0.000	-0.874	0.000	-0.375	0.000	-0.589	0.000	-0.501	0.000	-0.403	0.000	-0.305	0.000
Systolic blood pressure (mmHg)		0.415	0.000	-0.454	0.000	-0.176	0.018	-0.249	0.001	-0.229	0.002	-0.120	0.136	-0.185	0.013
Diastolic blood pressure (mmHg)		0.474	0.000	-0.505	0.000	-0.149	0.046	-0.219	0.003	-0.238	0.001	-0.140	0.061	-0.207	0.005
Fasting plasma glucose (mg/dL)		0.307	0.000	-0.353	0.000	-0.219	0.003	-0.141	0.059	-0.170	0.023	-0.104	0.167	-0.076	0.313
HbA1c (%)		0.408	0.000	-0.363	0.000	-0.197	0.008	-0.202	0.006	-0.138	0.064	-0.138	0.064	-0.159	0.033
Creatinine (mg/dL)		-0.167	0.025	0.195	0.009	0.309	0.000	0.114	0.127	0.341	0.000	0.199	0.008	0.256	0.001
Alanine aminotransferase (IU/L)		0.370	0.000	-0.340	0.000	-0.101	0.176	-0.239	0.001	-0.116	0.123	-0.175	0.019	-0.279	0.000
Total cholesterol (mg/dL)		0.076	0.314	-0.063	0.399	0.010	0.890	-0.005	0.945	0.024	0.748	0.076	0.310	-0.067	0.371
Triglyceride (mg/dL)		0.325	0.000	-0.319	0.000	-0.104	0.165	-0.144	0.054	-0.147	0.049	-0.101	0.179	-0.134	0.073
High-density lipoprotein-cholesterol (mg/dL)		-0.468	0.000	0.479	0.000	0.156	0.036	0.260	0.000	0.235	0.001	0.193	0.009	0.167	0.025
Glucose tolerance test															
Plasma glucose (mg/dL)	0 min	0.307	0.000	-0.353	0.000	-0.219	0.003	-0.141	0.059	-0.170	0.023	-0.104	0.167	-0.076	0.313
	30 min	0.135	0.072	-0.156	0.037	-0.053	0.478	-0.058	0.440	-0.051	0.497	-0.024	0.753	-0.052	0.489
	60 min	0.330	0.000	-0.320	0.000	-0.170	0.022	-0.224	0.003	-0.172	0.021	-0.154	0.039	-0.088	0.239
	120 min	0.318	0.000	-0.298	0.000	-0.155	0.038	-0.140	0.061	-0.191	0.010	-0.117	0.118	-0.166	0.026
Serum insulin (IU/L)	0 min	0.581	0.000	-0.585	0.000	-0.233	0.002	-0.331	0.000	-0.345	0.000	-0.312	0.000	-0.343	0.000
	30 min	0.271	0.000	-0.305	0.000	-0.110	0.140	-0.206	0.006	-0.242	0.001	-0.173	0.021	-0.256	0.001
	60 min	0.407	0.000	-0.417	0.000	-0.217	0.003	-0.294	0.000	-0.287	0.000	-0.270	0.000	-0.296	0.000
	120 min	0.466	0.000	-0.453	0.000	-0.183	0.014	-0.291	0.000	-0.307	0.000	-0.217	0.004	-0.316	0.000
Insulinogenic index		0.128	0.087	-0.152	0.042	-0.104	0.163	-0.139	0.063	-0.163	0.029	-0.121	0.106	-0.170	0.023
Hepatic insulin resistance index		0.360	0.000	-0.397	0.000	-0.156	0.037	-0.235	0.001	-0.281	0.000	-0.200	0.007	-0.284	0.000
Matsuda index		-0.600	0.000	0.612	0.000	0.271	0.000	0.363	0.000	0.381	0.000	0.322	0.000	0.373	0.000
HOMA-IR		0.592	0.000	-0.603	0.000	-0.252	0.001	-0.332	0.000	-0.350	0.000	-0.309	0.000	-0.334	0.000
QUICKI		-0.590	0.000	0.599	0.000	0.247	0.001	0.333	0.000	0.347	0.000	0.298	0.000	0.327	0.000
Partial correlation was analysed, adjusted with age and sex.

Similarly, all the weight-adjusted sectional areas of specific skeletal muscles showed a stronger correlation with metabolic parameters (Table 3A, Fig. 2A, Fig. 2B). However, the correlation coefficients were lower than those of the weight-adjusted lean body mass.

Because 34 out of 195 subjects were taking antihypertensive and/or lipid-lowering agents, same analyses were performed by excluding subjects taking antihypertensive (Table 3B) and lipid-lowering (Table 3C) agents. Similar results, showing weight-adjusted fat/lean body mass or weight-adjusted computed tomography-assessed maximal sectional area of specific skeletal muscles with metabolic parameters, were obtained when subjects taking antihypertensive and lipid-lowering agents were excluded.

**Table 3B:** Partial correlation of weight-adjusted fat mass, lean body mass, and sectional area of specific skeletal muscles with metabolic parameters in subjects not taking anti-hypertension drugs.

			Weight-adjusted fat mass		Weight-adjusted lean body mass		Weight-adjusted iliopsoas muscle		Weight-adjusted erector spinae muscle		Weight-adjusted gluteal muscle		Weight-adjusted femoris muscle		Weight-adjusted rectus abdominis muscle	
			r	p	r	p	r	p	r	p	r	p	r	p	r	p
Waist circumference (cm)			0.845	0.000	-0.875	0.000	-0.369	0.000	-0.611	0.000	-0.462	0.000	-0.389	0.000	-0.330	0.000
Systolic blood pressure (mmHg)			0.422	0.000	-0.455	0.000	-0.199	0.013	-0.246	0.002	-0.192	0.017	-0.077	0.340	-0.193	0.016
Diastolic blood pressure (mmHg)			0.481	0.000	-0.506	0.000	-0.155	0.054	-0.213	0.008	-0.206	0.010	-0.131	0.104	-0.235	0.003
Fasting plasma glucose (mg/dl)			0.285	0.000	-0.338	0.000	-0.219	0.006	-0.205	0.011	-0.149	0.065	-0.073	0.369	-0.071	0.380
HbA1c (%)			0.383	0.000	-0.336	0.000	-0.207	0.010	-0.290	0.000	-0.108	0.179	-0.112	0.167	-0.151	0.061
Creatinine (mg/dl)			-0.198	0.013	0.241	0.002	0.344	0.000	0.162	0.044	0.380	0.000	0.225	0.005	0.269	0.001
Alanine aminotransferase (IU/L)			0.380	0.000	-0.355	0.000	-0.163	0.042	-0.261	0.001	-0.155	0.054	-0.174	0.030	-0.246	0.002
Total cholesterol (mg/dL)			0.124	0.125	-0.121	0.134	-0.018	0.822	-0.051	0.530	-0.014	0.861	0.043	0.596	-0.121	0.133
Triglyceride (mg/dL)			0.320	0.000	-0.317	0.000	-0.111	0.171	-0.146	0.069	-0.114	0.157	-0.102	0.208	-0.139	0.085
High-density lipoprotein-cholesterol (mg/dL)			-0.465	0.000	0.482	0.000	0.146	0.070	0.287	0.000	0.189	0.018	0.176	0.028	0.141	0.081
Glucose tolerance test																
	Plasma glucose (mg/dl)	0 min	0.285	0.000	-0.338	0.000	-0.219	0.006	-0.205	0.011	-0.149	0.065	-0.073	0.369	-0.071	0.380
		30 min	0.106	0.191	-0.136	0.091	-0.066	0.414	-0.109	0.177	-0.058	0.473	-0.022	0.782	-0.044	0.584
		60 min	0.304	0.000	-0.297	0.000	-0.165	0.040	-0.241	0.003	-0.159	0.048	-0.014	0.094	-0.087	0.283
		120 min	0.278	0.000	-0.261	0.001	-0.122	0.129	-0.145	0.072	-0.143	0.077	-0.063	0.436	-0.130	0.107
	Serum insulin (IU/L)	0 min	0.571	0.000	-0.579	0.000	-0.255	0.001	-0.375	0.000	-0.349	0.000	-0.303	0.000	-0.303	0.000
		30 min	0.264	0.001	-0.303	0.000	-0.148	0.066	-0.214	0.007	-0.249	0.002	-0.198	0.014	-0.213	0.008
		60 min	0.396	0.000	-0.410	0.000	-0.288	0.000	-0.313	0.000	-0.310	0.000	-0.298	0.000	-0.268	0.001
		120 min	0.468	0.000	-0.457	0.000	-0.229	0.004	-0.291	0.000	-0.315	0.000	-0.212	0.001	-0.279	0.000
Insulinogenic index			0.131	0.105	-0.150	0.063	-0.117	0.148	-0.105	0.194	-0.146	0.070	-0.132	0.101	-0.142	0.079
Hepatic insulin resistance index			0.345	0.000	-0.391	0.000	-0.192	0.017	-0.267	0.001	-0.287	0.000	-0.217	0.007	-0.237	0.003
Matsuda index			-0.598	0.000	0.618	0.000	0.319	0.000	0.416	0.000	0.397	0.000	0.328	0.000	0.336	0.000
HOMA-IR			0.584	0.000	-0.600	0.000	-0.274	0.001	-0.386	0.000	-0.354	0.000	-0.299	0.000	-0.298	0.000
QUICKI			-0.580	0.000	0.593	0.000	0.267	0.001	0.382	0.000	-0.350	0.000	0.288	0.000	0.295	0.000
Partial correlation was analysed, adjusted with age and sex.

**Table 3C:** Partial correlation of weight-adjusted fat mass, lean body mass, and sectional area of specific skeletal muscles with metabolic parameters in subjects not taking anti-hyperlipidemia drugs.

			Weight-adjusted fat mass		Weight-adjusted lean body mass		Weight-adjusted iliopsoas muscle		Weight-adjusted erector spinae muscle		Weight-adjusted gluteal muscle		Weight-adjusted femoris muscle		Weight-adjusted rectus abdominis muscle	
			r	p	r	p	r	p	r	p	r	p	r	p	r	p
Waist circumference (cm)			0.844	0.000	-0.872	0.000	-0.351	0.000	-0.584	0.000	-0.468	0.000	-0.379	0.000	-0.269	0.001
Systolic blood pressure (mmHg)			0.435	0.000	-0.468	0.000	-0.146	0.063	-0.276	0.000	-0.197	0.012	-0.102	0.194	-0.159	0.043
Diastolic blood pressure (mmHg)			0.480	0.000	-0.505	0.000	-0.115	0.140	-0.231	0.003	-0.202	0.010	-0.137	0.081	-0.207	0.008
Fasting plasma glucose (mg/dL)			0.273	0.000	-0.316	0.000	-0.187	0.017	-0.114	0.146	-0.112	0.153	-0.059	0.457	-0.049	0.531
HbA1c (%)			0.380	0.000	-0.329	0.000	-0.204	0.009	-0.192	0.014	-0.112	0.156	-0.096	0.225	-0.097	0.220
Creatinine (mg/dL)			-0.177	0.024	0.211	0.007	0.313	0.000	0.128	0.103	0.353	0.000	0.215	0.006	0.292	0.000
Alanine aminotransferase (IU/L)			0.365	0.000	-0.346	0.000	-0.132	0.092	-0.255	0.001	-0.134	0.088	-0.148	0.059	-0.224	0.004
Total cholesterol (mg/dL)			0.099	0.209	-0.093	0.240	-0.008	0.921	-0.011	0.885	0.019	0.810	0.072	0.363	-0.078	0.323
Triglyceride (mg/dL)			0.316	0.000	-0.312	0.000	-0.094	0.231	-0.137	0.082	-0.113	0.153	-0.089	0.260	-0.110	0.161
High-density lipoprotein-cholesterol (mg/dL)			-0.453	0.000	0.468	0.000	0.134	0.087	0.251	0.001	0.213	0.006	0.157	0.045	0.124	0.115
Glucose tolerance test																
	Plasma glucose (mg/dL)	0 min	0.273	0.000	-0.316	0.000	-0.187	0.017	-0.114	0.146	-0.112	0.153	-0.059	0.457	-0.049	0.531
		30 min	0.119	0.129	-0.145	0.064	-0.067	0.394	-0.064	0.416	-0.049	0.537	-0.004	0.964	-0.012	0.875
		60 min	0.311	0.000	-0.305	0.000	-0.177	0.024	-0.210	0.007	-0.162	0.039	-0.134	0.089	-0.050	0.529
		120 min	0.294	0.000	-0.272	0.000	-0.161	0.040	-0.115	0.143	-0.182	0.020	-0.072	0.359	-0.105	0.182
	Serum insulin (IU/L)	0 min	0.556	0.000	-0.565	0.000	-0.217	0.005	-0.342	0.000	-0.326	0.000	-0.291	0.000	-0.304	0.000
		30 min	0.253	0.000	-0.292	0.000	-0.095	0.225	-0.229	0.003	-0.226	0.004	-0.158	0.044	-0.207	0.008
		60 min	0.391	0.000	-0.407	0.000	-0.225	0.004	-0.307	0.000	-0.280	0.000	-0.262	0.001	-0.247	0.001
		120 min	0.462	0.000	-0.452	0.000	-0.201	0.010	-0.303	0.000	-0.319	0.000	-0.192	0.014	-0.249	0.001
Insulinogenic index			0.114	0.146	-0.135	0.086	-0.068	0.390	-0.142	0.070	-0.125	0.111	-0.110	0.163	-0.148	0.059
Hepatic insulin resistance index			0.338	0.000	-0.381	0.000	-0.143	0.069	-0.257	0.001	-0.262	0.001	-0.178	0.023	-0.227	0.004
Matsuda index			-0.582	0.000	0.600	0.000	0.267	0.001	0.380	0.000	0.369	0.000	0.298	0.000	0.318	0.000
HOMA-IR			0.565	0.000	-0.580	0.000	-0.233	0.003	-0.339	0.000	-0.324	0.000	-0.283	0.000	-0.294	0.000
QUICKI			-0.562	0.000	0.575	0.000	0.227	0.004	0.337	0.000	0.321	0.000	0.272	0.000	0.289	0.000
Partial correlation was analysed, adjusted with age and sex.																

### 3.3 Anthropometric measures reflecting lean body and metabolic parameters

Because it is relatively difficult to measure lean body mass in the clinical setting and during a routine health examination, a conventional anthropometric measure that best reflects the lean body mass is important. Therefore, we examined anthropometric measures that may reflect body composition parameters in each gender.

We determined inter-investigator variations among four of 6 nurses who participated in the present study in the measurement of all of the anthropometric indices. Four nurses independently measured AC, TC, WC, HC, and CC of 10 independent test subjects. Intraclass correlation coefficients (95% CI) were calculated in each index as follows; 0.996 (0.915–0.990) in AC, 0.990 (0.966–0.997) in WC, 0.974 (CI 0.902–0.993) in HC, 0.988 (0.971–0.997) in TC, and 0.994 (0.982–0.999) in CC.

**Fig. 3:**
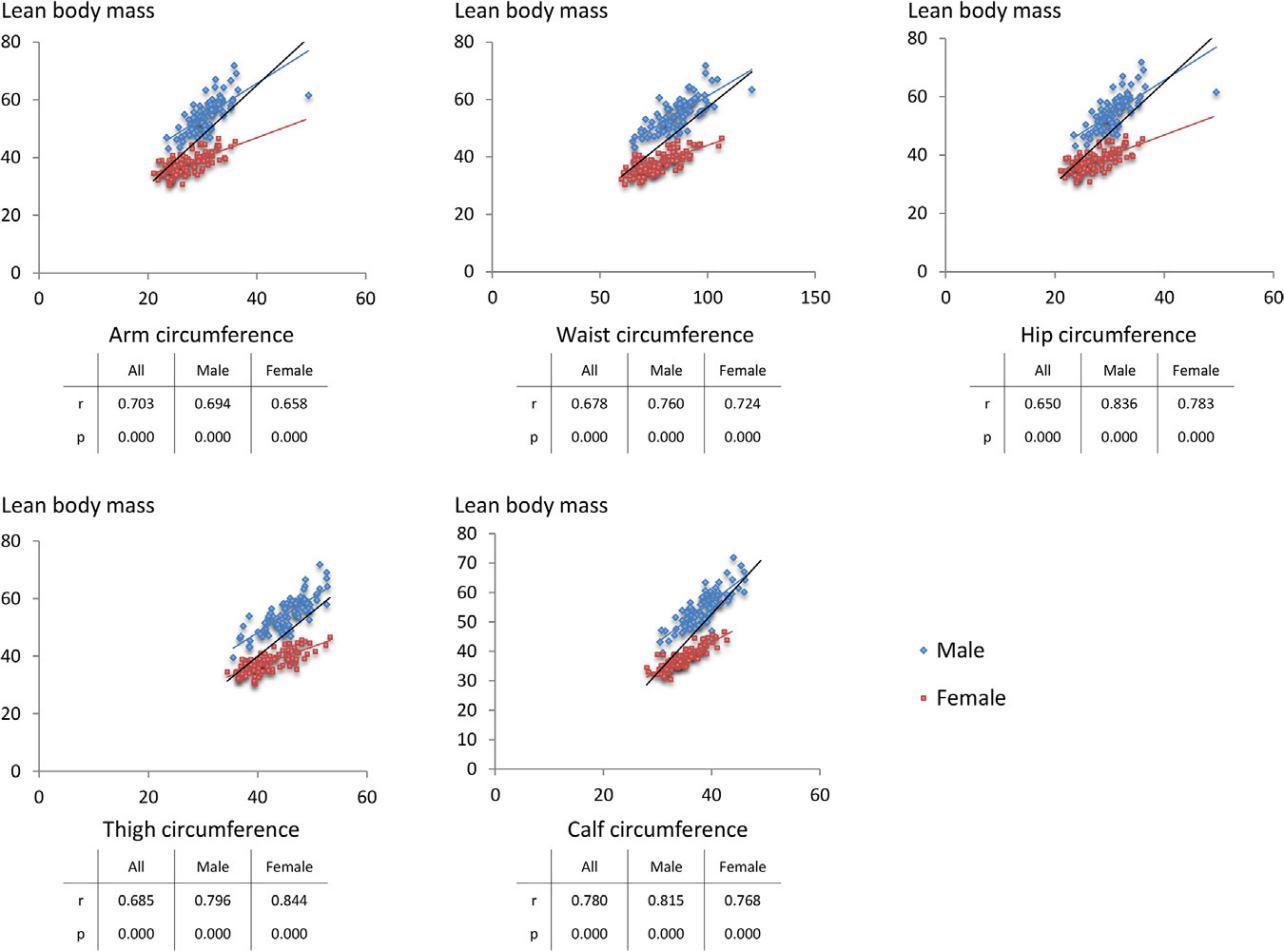
Scatter diagrams between lean body mass and various circumferences.

**Fig. 4:**
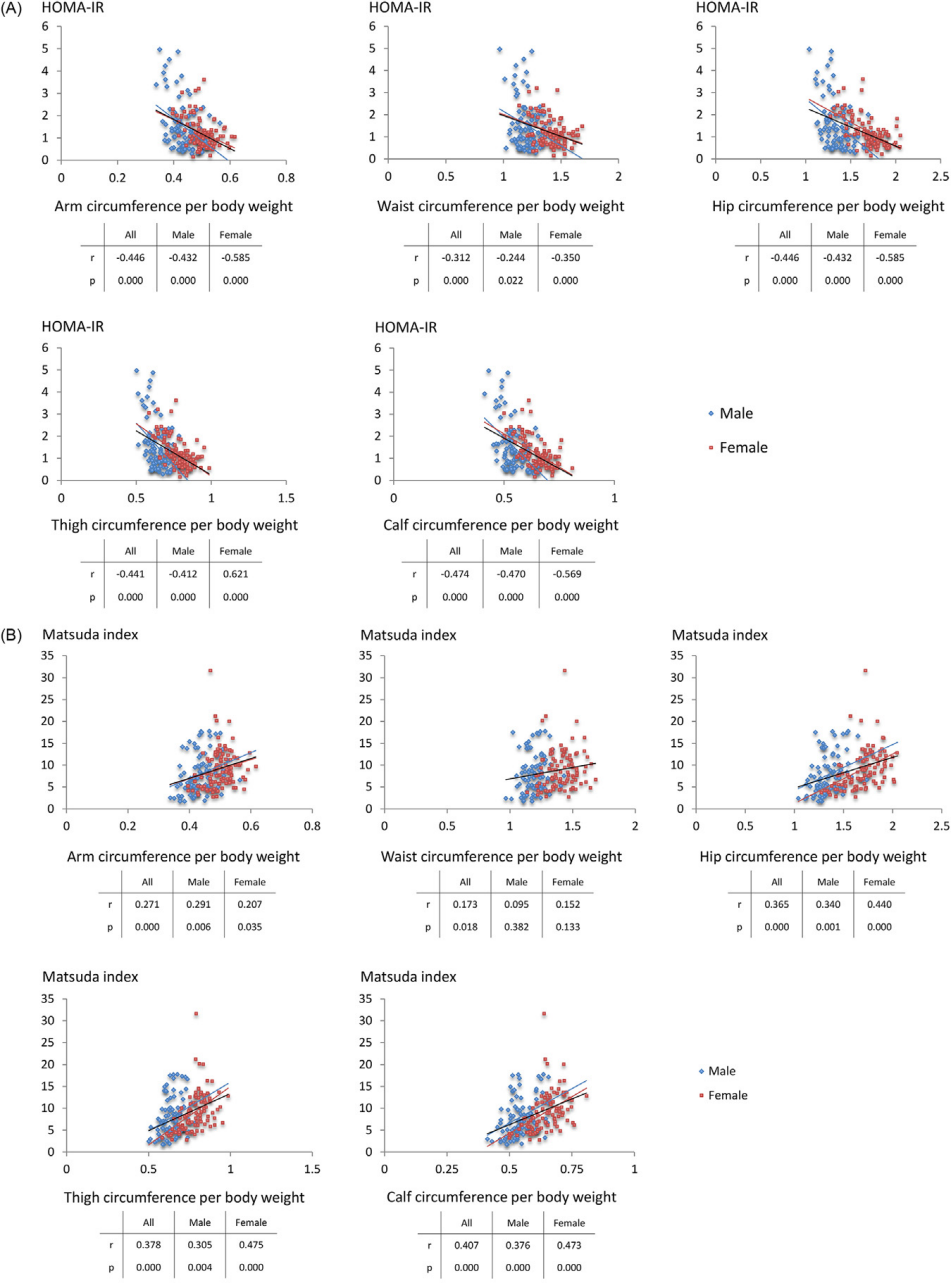
A. Scatter diagrams between insulin resistance HOMA-IR index and various circumferences per body weight. B. Scatter diagrams between insulin sensitivity Matsuda index and various circumferences per body weight.

In a univariate correlation analyses, all anthropometric measures evaluated (AC, WC, HC, TC, and CC) positively correlated with lean body mass in men and women (Table 4, Fig. 3). Furthermore, multiple linear regression analysis showed that the anthropometric measures that best reflect lean body mass in men and women were HC and CC, respectively, whereas TC was not an independent variable reflecting lean body mass in either gender (Table 5). When both genders were analyzed together, CC best reflected lean body mass (Table 5). As shown in Table 6, Fig. 4A, and Fig. 4B, similar to weight-adjusted lean body mass, weight-adjusted CC negatively correlated with systolic and diastolic blood pressure; fasting plasma glucose, HbA1c, alanine aminotransferase, TG, and insulin levels; and insulin resistance indices, such as H-IR and HOMA-IR (Fig. 4A), and positively correlated with HDL-C levels and insulin sensitivity indices, such as Matsuda index (Fig. 4B) and QUICKI.

**Table 4:** Univariate correlations of anthropometric measures with lean body mass.

	All		Male		Female	
	r	P	r	P	r	P
Arm circumference (cm)	0.703	0.000	0.694	0.000	0.668	0.000
Waist circumference (cm)	0.678	0.000	0.760	0.000	0.724	0.000
Hip circumference (cm)	0.650	0.000	0.837	0.000	0.782	0.000
Thigh circumference (cm)	0.689	0.000	0.785	0.000	0.765	0.000
Calf circumference (cm)	0.779	0.000	0.814	0.000	0.844	0.000

**Table 5:** Multiple linear regression analysis showing variables independently associated with lean body mass.

	All			Male			Female		
	Coefficient (β)	t-statistic	P	Coefficient (β)	t-statistic	P	Coefficient (β)	t-statistic	P
Arm circumference (cm)	0.222	2.564	0.011	0.200	2.446	0.017	-0.230	-2.214	0.029
Waist circumference (cm)	0.183	1.605	0.110	-0.227	-1.656	0.102	0.100	0.876	0.383
Hip circumference (cm)	-0.154	-1.298	0.196	0.627	4.405	0.000	2.451	2.451	0.016
Thigh circumference (cm)	-0.149	-1.352	0.178	0.056	0.465	0.643	-0.029	-0.248	0.805
Calf circumference (cm)	0.718	6.736	0.000	0.306	2.874	0.005	0.714	5.778	0.000
Multiple linear regression was used for the analysis, adjusted with each other.

**Table 6:** Partial correlation of weight-adjusted various circumference with metabolic parameters.

			Weight-adjusted arm circumferance		Weight-adjusted waist circumferance		Weight-adjusted hip circumferance		Weight-adjusted thigh circumferance		Weight-adjusted calf circumferance	
			r	p	r	p	r	p	r	p	r	p
Body weight (kg)			-0.731	0.000	-0.871	0.000	-0.941	0.000	-0.855	0.000	-0.925	0.000
Systolic blood pressure (mmHg)			-0.214	0.004	-0.247	0.001	-0.387	0.000	-0.355	0.000	-0.467	0.000
Diastolic blood pressure (mmHg)			-0.314	0.000	-0.312	0.000	-0.468	0.000	-0.439	0.000	-0.563	0.000
Fasting plasma glucose (mg/dL)			-0.261	0.000	-0.171	0.021	-0.292	0.000	-0.289	0.000	-0.463	0.000
HbA1c (%)			-0.274	0.000	-0.158	0.033	-0.312	0.000	-0.363	0.000	-0.333	0.000
Creatinine (mg/dL)			0.166	0.025	0.014	0.850	0.098	0.190	0.128	0.086	-0.437	0.000
Alanine aminotransferase (IU/L)			-0.240	0.001	-0.246	0.001	-0.339	0.000	-0.304	0.000	-0.483	0.000
Total cholesterol (mg/dL)			-0.045	0.543	-0.045	0.546	-0.086	0.246	-0.105	0.158	0.018	0.802
Triglyceride (mg/dL)			-0.051	0.497	-0.055	0.461	-0.139	0.062	-0.133	0.074	-0.234	0.001
High-density lipoprotein-cholesterol (mg/dL)			0.247	0.001	0.219	0.003	0.412	0.000	0.349	0.000	0.511	0.000
Glucose tolerance test												
	Plasma glucose (mg/dL)	0 min	-0.261	0.000	-0.171	0.021	-0.292	0.000	-0.289	0.000	-0.463	0.000
		30 min	-0.053	0.473	0.086	0.249	-0.065	0.380	-0.114	0.125	-0.368	0.066
		60 min	-0.172	0.020	-0.012	0.872	-0.223	0.002	-0.285	0.000	-0.463	0.000
		120 min	-0.185	0.013	-0.125	0.094	-0.244	0.001	-0.172	0.020	-0.341	0.000
	Serum insulin (IU/L)	0 min	-0.371	0.000	-0.274	0.000	-0.476	0.000	-0.457	0.000	-0.437	0.000
		30 min	-0.152	0.041	-0.126	0.089	-0.255	0.001	-0.207	0.005	-0.132	0.000
		60 min	-0.224	0.002	-0.093	0.211	-0.279	0.000	-0.347	0.000	-0.332	0.000
		120 min	-0.181	0.014	-0.136	0.067	-0.270	0.000	-0.257	0.000	-0.333	0.000
Insulinogenic index			-0.085	0.254	-0.126	0.091	-0.174	0.019	-0.126	0.089	0.034	0.641
Hepatic insulin resistance index			-0.207	0.005	-0.148	0.046	-0.307	0.000	-0.268	0.000	-0.279	0.000
Matsuda index			0.258	0.000	0.148	0.046	0.382	0.000	0.390	0.000	0.407	0.000
HOMA-IR			-0.388	0.000	-0.278	0.000	-0.479	0.000	-0.462	0.000	-0.474	0.000
QUICKI			0.324	0.000	0.228	0.002	0.437	0.000	0.413	0.000	0.407	0.000
Partial correlation was analysed, adjusted with age and sex.

## 4 Discussion

The present study identified weight-adjusted lean body mass and skeletal muscle areas as indicators of systemic insulin sensitivity and protective against weight-associated metabolic abnormalities in subjects without diabetes. Interestingly, fat and lean body mass are both significantly associated with insulin resistance and metabolic abnormalities, suggesting that not only fat mass but also absolute lean body mass increases in subjects with obesity. After adjusting for weight, the relationships of fat and lean body mass with metabolic parameters became mirror images. These findings suggest that the proportion of the skeletal muscle, rather than the absolute skeletal muscle mass, may be used as a protective measure against obesity-associated insulin resistance and metabolic abnormalities.

In the present study, we determined the sectional areas of specific skeletal muscles, such as the iliopsoas, rector spinae, gluteus, femoris, and rectus abdominis muscle, using CT. Similar to the weight-adjusted lean body mass, all weight-adjusted sectional areas of each specific skeletal muscle showed a positive correlation with insulin resistance and metabolic abnormalities. However, unexpectedly, lean body mass had a stronger impact on insulin sensitivity and energy metabolism than the sectional area of specific skeletal muscles. Although we cannot rule out the possibility that CT-assessed skeletal muscle mass, rather than maximal area of each skeletal muscle, has a stronger impact on insulin sensitivity and energy metabolism, weight-adjusted lean body mass appears to be more useful for predicting protection against obesity-associated insulin resistance and metabolic abnormalities.

The loss of muscle mass is a consequence of physical inactivity. In response to physical activity, muscles release bioactive peptides, namely, myokines, which stimulate muscle growth and hypertrophy, enhance insulin sensitivity, and thereby, protect against obesity-associated metabolic abnormalities [2]. Based on these findings, we conclude that lean body mass, and possibly skeletal muscle mass, are protective against weight-associated insulin resistance and metabolic abnormalities.

Unfortunately, estimating lean body mass requires an electrical impedance method or dual energy X-ray absorptiometry. Therefore, to apply our findings to routine health examinations in the average clinical setting, we identified an anthropometric parameter, CC, that reflects the lean body mass in both men and women. Similar to the weight-adjusted lean body mass, weight-adjusted CC in both men and women was also protective against insulin resistance and metabolic abnormalities. CC has been used as an index of nutritional state and weight of bedridden elderly patients [13]. Furthermore, an association of CC with insulin resistance and carotid atherosclerosis was reported [14]. Recently, we found that lean body mass and CC are associated with basal energy expenditure and partly with diet-induced thermogenesis in patients with diabetes [6]. Together with the present findings, we propose to measure CC to estimate lean body mass and the potential protection against obesity-associated insulin resistance and metabolic abnormalities during routine health examinations. Measuring CC may also be useful as an indicator of systemic skeletal muscle mass when starting exercise therapy at the hospital or fitness club.

Limitations in the present study were as follows. First, 34 out of 195 subjects were taking antihypertensive and/or lipid-lowering agents. However, similar results, showing fat/lean body mass or computed tomography-assessed maximal sectional area of specific skeletal muscles with metabolic parameters, were obtained when subjects taking antihypertensive (Table 2B) and lipid-lowering (Table 2C) agents were excluded. Second, we did not estimate fitness level or circulating levels of myokines, which may affect skeletal muscle mass and metabolic outcomes. Third, the sample size was not large enough to perform intensive subanalyses. A large-scale prospective cohort study is needed to determine set points and desired values for primary and secondary preventions against lifestyle-related diseases, such as obesity, type 2 diabetes, and cardiovascular diseases. Also, a prospective intervention study is required to confirm that increased fat-free mass and CC may protect against obesity and related metabolic abnormalities.

In conclusion, weight-adjusted lean body mass and skeletal muscle areas are protective against weight-associated insulin resistance and metabolic abnormalities. Among anthropometric measures, CC best reflects the lean body mass in both men and women and may be useful as a protective marker against obesity-associated metabolic abnormalities. In future health examinations, in addition to WC, CC might be used as an index of skeletal muscle mass and as a protective marker against obesity-associated metabolic abnormalities.

## Declarations

### Author contribution statement

Toshinari Takamura: Conceived and designed the experiments; Analyzed and interpreted the data; Wrote the paper.

Masatoshi Nakagen, Kohzo Kawai, Takeshi Urabe: Performed the experiments; Contributed reagents, materials, analysis tools or data.

Yuki Kita: Performed the experiments; Analyzed and interpreted the data; Contributed reagents, materials, analysis tools or data.

Masaru Sakurai, Yuki Isobe, Yumie Takeshita: Analyzed and interpreted the data.

Shuichi Kaneko: Conceived and designed the experiments.

### Funding statement

This research did not receive any specific grant from funding agencies in the public, commercial, or not-for-profit sectors.

### Competing interest statement

The authors declare no conflict of interest.

### Additional information

The clinical trial described in this paper was registered at University Hospital Medical Information Network (UMIN) Clinical Trials Registry under the registration number UMIN000012630.
