# Corrigendum to ‘Weight-adjusted lean body mass and calf circumference are protective against obesity-associated insulin resistance and metabolic abnormalities’

**DOI:** 10.1016/j.heliyon.2017.e00363

**Published:** 2017-07-26

**Authors:** Toshinari Takamura, Yuki Kita, Masatoshi Nakagen, Masaru Sakurai, Yuki Isobe, Yumie Takeshita, Kohzo Kawai, Takeshi Urabe, Shuichi Kaneko

**Affiliations:** a Department of Endocrinology and Metabolism, Kanazawa University Graduate School of Medical Sciences, Ishikawa, Japan; b Department of System Biology, Kanazawa University Graduate School of Medical Sciences, Ishikawa, Japan; c Social and Environmental Medicine, Kanazawa Medical University, Ishikawa, Japan; d Department of Internal Medicine, Public Central Hospital of Matto Ishikawa, Hakusan, Ishikawa, Japan

In the original published version of this article, a typographical error was present in Table 5 displaying ‘2.451′ in ‘Column: Female, Coefficient (β); Row: Hip Circumference’ instead of the correct value of ‘0.322′. The authors apologize for the mistake. The corrected version of Table 5 is displayed below.

**Table 5:** Multiple linear regression analysis showing variables independently associated with lean body mass.

	All			Male			Female		
	Coefficient (β)	t-statistic	P	Coefficient (β)	t-statistic	P	Coefficient (β)	t-statistic	P
Arm circumference (cm)	0.222	2.564	0.011	0.200	2.446	0.017	−0.230	−2.214	0.029
Waist circumference (cm)	0.183	1.605	0.110	−0.227	−1.656	0.102	0.100	0.876	0.383
Hip circumference (cm)	−0.154	−1.298	0.196	0.627	4.405	0.000	0.322	2.451	0.016
Thigh circumference (cm)	−0.149	−1.352	0.178	0.056	0.465	0.643	−0.029	−0.248	0.805
Calf circumference (cm)	0.718	6.736	0.000	0.306	2.874	0.005	0.714	5.778	0.000

Multiple linear regression was used for the analysis, adjusted with each other.

